# Tumour Microenvironment-Informed Radiotheranostics: Why and How Nuclear Medicine Could Advance Precision Oncology in the Decade Ahead

**DOI:** 10.3390/ph19030382

**Published:** 2026-02-27

**Authors:** Kalyani Pandya, Zhaoguo Lin, Magdalena Wadsak, Jiahui Wang, Kuangyu Shi, Robert Seifert

**Affiliations:** 1 Department of Nuclear Medicine, Inselspital, University Hospital Bern, University of Bern, 3008 Bern, Switzerland; zhaoguo.lin@students.unibe.ch (Z.L.); magdalena.wadsak@students.unibe.ch (M.W.); jiahui.wang@students.unibe.ch (J.W.); kuangyu.shi@unibe.ch (K.S.); 2 Department of Nuclear Medicine, Union Hospital, Tongji Medical College, Huazhong University of Science and Technology, Wuhan 430022, China; 3 Hubei Key Laboratory of Molecular Imaging, Wuhan 430022, China

**Keywords:** theranostics, PET, oncology, tumour microenvironment

## Abstract

Despite significant progress in targeted cancer therapies and conventional imaging methodologies, the effective detection and treatment of solid tumours remain a major clinical challenge. This is thought to be caused by the complexity and heterogeneity found in the tumour microenvironment (TME), which significantly effects drug delivery and therapeutic response. Different levels of fibrosis, varying immune-cell infiltration, and disorganized vasculature form barriers for therapeutic approaches. However, in the next decade, radiotheranostics, defined here as the combined use of matched diagnostic and therapeutic radiopharmaceuticals, could present a targeted and flexible strategy for addressing some of the challenges caused by the TME. By combining molecular imaging with therapeutic delivery, it enables the in vivo visualization of TME features and the selective treatment of tumour and stromal compartments. This provides the unique opportunity to target tumour regions resistant to conventional therapies, including those shaped by (extracellular matrix) ECM stiffness, immune infiltration, or hypoxia. However, new strategies are needed to identify targets and evaluate their efficacy for more precise therapies. In this review, we will discuss why radiotheranostics is an ideal field for advancing the therapeutic approaches to solid tumours by incorporating the growing understanding of the TME. We will discuss how key microenvironmental features affect radiotracer distribution and treatment outcomes. We will highlight emerging tools including ECM- and immune-targeted imaging, patient-derived organoids, and organ-on-chip models which will be instrumental in developing physiologically relevant radiopharmaceutical therapies. Finally, we will discuss how spatial/single-cell transcriptomic approaches can support target discovery and allow for patient outcome assessment, with the aim of integrating microenvironment-aware insights into the development of novel radiotheranostic agents.

## 1. Introduction

In solid tumours, the tumour microenvironment (TME) critically shapes diagnostic accuracy and therapeutic efficacy, bridging the gap between tumour biology heterogeneity and cellular makeup. TME-regulating mechanisms promote tumour cell survival by enhancing proliferation, evading cell death, avoiding immune recognition, and facilitating metastasis [1,2,3]. This heterogeneity, together with the absence of well-defined structures and the presence of abnormal tissue proliferation, makes solid tumours especially challenging to treat [4,5,6]. As a result, current management approaches including surgical resection, radiotherapy, and chemotherapy frequently lead to recurrence, particularly for the latter two options, where TME-mediated resistance proves decisive [7,8].

Radiotheranostics directly addresses these challenges by integrating diagnostic imaging with targeted radiopharmaceutical therapy (RPT), enabling TME-informed personalization [9]. Emerging evidence confirms that stromal organization, immune composition, and vascular function directly modulate radiopharmaceutical distribution and efficacy [10,11].

This review examines how TME barriers impair radiotheranostic success and presents microenvironment-responsive strategies—from target selection to tracer design—that can overcome these obstacles to transform precision oncology. Together, these developments outline why radiotheranostics is poised to become increasingly important over the next decade: it uniquely integrates biological insight from the TME with precision delivery of therapeutic radiation. As these capabilities mature, radiotheranostics will be positioned to address therapeutic resistance that conventional therapies cannot overcome.

## 2. The TME as a Barrier to Radiotheranostics

**Figure 1:**
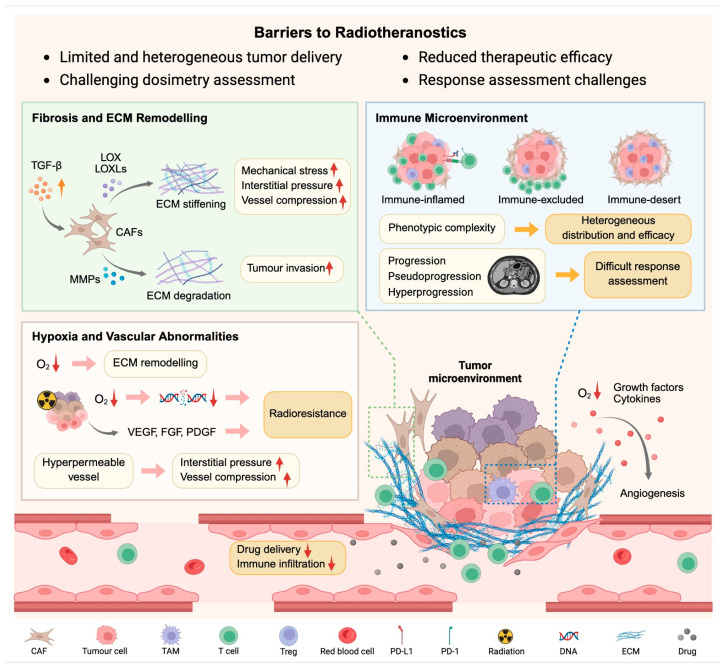
Schematic of TME barriers (fibrosis/ECM, immune infiltration, vasculature/hypoxia) and their impact on radiotheranostics. Fibrosis impairs delivery and immune access; immune exclusion limits tracer efficacy and confounds response assessment; hypoxia/vascular dysfunction induces radioresistance. These components interact dynamically to hinder radiotheranostic success. Abbreviations: CAF, cancer-associated fibroblast; ECM, extracellular matrix; LOX/LOXLs, lysyl oxidase/LOXLs; MMP, matrix metalloproteinase; PD-1/PD-L1, programmed death protein-1/ligand 1; TAM, tumour-associated macrophage; TGF-β, transforming growth factor-β; VEGF, vascular endothelial growth factor. Created in BioRender. Lin, Z. (2026) https://BioRender.com/b47aeqw, accessed on 22 February 2026.

The TME comprises physical, mechanical, metabolic, inflammatory, and immune elements that impede radiopharmaceutical access and efficacy [3]. This section focuses on three key barriers—ECM/fibrosis, immune exclusion, and vascular dysfunction—most relevant to radiotheranostics (Figure 1).

### 2.1. Fibrosis and Extracellular Matrix (ECM) Remodelling

The ECM is composed of hundreds of macromolecules which form a complex system [12]. It provides mechanical support and delivers essential biochemical and physical cues that regulate tissue development, differentiation, and homeostasis [12]. Structurally, it consists of the interstitial matrix, which is rich in collagens, fibronectin, elastin and the basement membrane, mainly composed of collagen IV and laminins linked by nidogen, perlecan, and heparan-sulphate proteoglycans [13].

Within the TME, CAFs reshape the interstitial ECM [14]. Activated mainly by TGF-β signalling, myofibroblastic CAFs (myCAFs) are the predominant source of ECM proteins [15]. Through the continuous synthesis, degradation, and remodelling of matrix components, CAFs dynamically reorganize the structural and mechanical landscape of tumours. They secrete proteolytic enzymes, including matrix metalloproteinases (MMP1, MMP9), to degrade and remodel the existing matrix, while exerting integrin-mediated contractile forces that align and compact collagen fibres [16]. CAFs also secrete crosslinking enzymes such as lysyl oxidases (LOX and LOXLs), which catalyze collagen crosslinking and increase matrix stiffness, thereby reinforcing tumour-associated fibrosis [17]. These coordinated processes promote ECM turnover, structural anisotropy, and mechanical tension, thereby facilitating cancer cell invasion, migration, and metastatic dissemination.

However, this ECM remodelling activates integrin-dependent signalling and releases sequestered growth factors/cytokines, while creating physical barriers to theranostics [18]. Increased matrix stiffness and elevated interstitial pressure compress vessels, impair perfusion, limit immune infiltration, and hinder both imaging tracers and therapeutic agents, yielding heterogeneous intra-tumoural distribution and reduced RPT efficacy [19]. In this context, collagen-targeted PET tracers such as ^68^Ga-NODAGAcollagelin, ^68^Ga-Collagen Binding Peptide (CBP) 8, enable in vivo quantification of fibrosis burden and response to antifibrotic therapies [20,21]. These tools could illustrate how stromal remodelling influences radiopharmaceutical delivery, with the potential to define impairment thresholds (e.g., fibrosis levels impairing linear tracer uptake) that remain incompletely understood [20]. However, the precise degree of fibrosis required to meaningfully impair tracer uptake—representing these impairment thresholds—remains incompletely understood due to limited research in this area, with the majority of collagen studies focusing on the detection of fibrosis only.

Therapeutic strategies targeting the ECM aim to modulate its structure and dynamics to overcome microenvironmental barriers. Current approaches include enzymatic degradation of matrix components (e.g., hyaluronidase [22]), inhibition of collagen crosslinking (e.g., LOXL2/LOXL3 blockade [23]), and CAF-directed interventions to restrain ECM remodelling [19]. In the field of radiotheranostics, fibroblast activation protein (FAP)-targeted radioligands leverage the abundance of CAFs within the TME and have shown promising preclinical and early clinical results [24]. ECM-binding radiopharmaceuticals directed against key matrix proteins such as tenascin-C and fibronectin have also been investigated [25,26,27,28], including radiolabelled aptamers, nanobodies, antibody fragments, and peptide-based probes, with applications ranging from PET and SPECT imaging to RPT. Most of the approaches have been validated in preclinical models, with only a subset advancing to clinical evaluation, and further studies are needed to substantiate their clinical efficacy.

### 2.2. Immune-Cell Infiltration and Immunosuppressive Niches

The TME comprises a heterogeneous immune landscape that includes T cells, natural killer cells, dendritic cells, and various myeloid cell populations. CD8^+^ cytotoxic T cells represent a primary effector of anti-tumour immunity and are essential for tumour cell elimination [29]. Tumour-associated macrophages (TAMs) and tumour-associated neutrophils (TANs) can have opposing roles, with M1-like/N1-like states promoting anti-tumour activity and M2-like/N2-like states facilitating angiogenesis, matrix remodelling, and immune suppression [30].

Based on the spatial distribution and activity of cytotoxic immune cells, tumours can be broadly categorized into three immunophenotypes [31]. Immune-inflamed (“hot”) tumours exhibit abundant intra-tumoural T cell infiltration, elevated interferon-γ signalling, and programmed death-ligand 1 (PD-L1) expression, often associated with favourable responses to immune-checkpoint inhibitors (ICIs). Immune-excluded tumours confine cytotoxic T cells to stromal or invasive margins due to physical and cellular barriers, and immune-desert tumours lack T cell infiltration altogether and are enriched with suppressive subsets such as TAMs, TANs, regulatory T cells, and myeloid-derived suppressor cells (MDSCs). The latter two phenotypes are generally considered “cold” tumours and show limited responsiveness to ICIs. The dynamic interplay among immune, stromal, and tumour components within the TME drives its continuous evolution and contributes to tumour heterogeneity [32]. Such heterogeneity increases the complexity of theranostic approaches, influencing both radiotracer uptake and treatment response.

For example, in imaging-based response assessment, this dynamic nature can lead to atypical patterns that challenge accurate interpretation. Pseudoprogression represents a transient increase in lesion size or metabolic activity caused by immune-cell infiltration and inflammation rather than true tumour growth, and hyperprogression denotes an abnormally rapid acceleration of disease following immunotherapy due to an immunosuppressive reprogramming of the TME [33]. Misclassification of pseudoprogression and hyperprogression may lead to premature discontinuation of effective therapy or delayed cessation of ineffective treatment. However, timely and accurate identification of them remains a major challenge in clinical practice.

In recent years, several dedicated imaging strategies have been developed to visualize immune activation within TME. Radiotracers targeting immune-checkpoint proteins, such as programmed cell death protein 1 (PD-1) and PD-L1, enable non-invasive visualization of their expression, facilitating the identification of patients who could show good response to ICIs [34,35]. Imaging of CD8^+^ T cells and functional markers of T cell activity, including granzyme B, interferon-γ (IFN-γ), and interleukin-2 (IL-2), has been explored for predicting or monitoring immunotherapy response [36,37,38,39,40]. Furthermore, probes targeting TAMs are under early-stage preclinical and clinical evaluation [41]. Beyond imaging applications, immune checkpoints such as PD-L1 and B7-H3 have also been exploited for therapeutic radionuclide delivery [42,43,44]. A ^177^Lu-labelled αPD-L1 antibody could alter the immune microenvironment within the TME, thereby synergistically enhancing the efficacy of immunotherapy [44,45]. An anti-PD-L1 single-domain antibody, ^177^Lu-RAD204, is being studied in a phase I trial in patients with metastatic solid tumours (NCT06305962). Radiolabelled antibodies directed against B7-H3, such as ^131^I-omburtamab and ^177^Lu-DTPA-omburtamab, have also entered phase I investigation [44,45].

### 2.3. Vascular Abnormalities and Hypoxia

Cancer cells are characterized by rapid proliferation and a highly active metabolism, leading to an increased demand for oxygen and nutrients. In the initial stages of tumour development, oxygen and nutrient supply mainly relies on diffusion from surrounding tissues [46]. This supply becomes insufficient once the tumour exceeds approximately 1 mm in diameter [47]. Consequently, regions of hypoxia and acidosis develop within the tumour mass. In response, tumour and stromal cells release several growth factors and cytokines that stimulate endothelial cell proliferation and migration, thereby initiating angiogenesis to meet the increased metabolic demands [48]. However, the neo-vasculature of tumours is often dysfunctional, leading to poor blood perfusion and inefficient oxygen delivery, which together exacerbate hypoxia within the TME [49].

Hypoxia and aberrant angiogenesis are hallmarks of most solid tumours and therefore partly responsible for tumour proliferation, invasion, metastasis, and therapeutic resistance [50,51]. The immature vasculature is characterized by discontinuous basement membranes, irregular endothelial alignment, and increased permeability [49]. This structural abnormality allows plasma and proteins to leak into the interstitium, leading to an increase in interstitial pressure and subsequent vessel compression, which together impair drug delivery [49]. Hypoxia also contributes to ECM remodelling by altering its composition, stimulating fibroblast proliferation and excessive matrix deposition, which increases mechanical stress and hinders drug delivery [52]. Apart from limiting drug transport, hypoxia and the vascular system also contribute to resistance to radiotherapy. Hypoxia can also diminish radiation efficacy by reducing oxygen-dependent induction of DNA damage [53].

With the widespread application of anti-angiogenic cancer therapeutics, radiopharmaceuticals have also been developed to target angiogenesis-related biomarkers such as integrins and VEGF receptors, allowing non-invasive assessment of their expression [54,55,56]. Regarding tumour hypoxia, several PET and SPECT tracers, including nitroimidazole-based compounds, have been designed to visualize tumour hypoxia [57]. Carbonic anhydrase IX (CAIX), a hypoxia-related cell-surface marker, is a promising target for radiotheranostics, particularly in clear-cell renal cell carcinoma (ccRCC), and is currently being investigated in early clinical studies [58,59].

These TME-driven barriers highlight why future progress in oncology will depend on radiotheranostic agents specifically engineered to overcome stromal, vascular, and immune obstacles that limit the success of targeted therapies.

Together, fibrosis, immune exclusion, and vascular dysfunction illustrate why TME-aware design is essential and motivate the delivery, off-target, and dosimetric challenges discussed in Section 3.

## 3. Barriers Imposed by the TME on Radiopharmaceutical Performance

This section translates the conceptual TME barriers outlined in Section 2 into four practical challenges for radiotheranostics: delivery and intra-tumoural distribution, off-target uptake, pharmacokinetics and clearance, and radiation dose and toxicity.

### 3.1. Delivery and Distribution Limitations

Effective radiotheranostic applications require adequate delivery of radiopharmaceuticals to malignant tissue. Yet, this process is hindered by TME barriers, particularly dense ECM from CAF activity and immune function (Section 2.1 and Section 2.2), which elevates interstitial pressure and restricts tracer/agent penetration [60]. Perfusion abnormalities described in Section 2.3 further amplify delivery limitations. Tumour vasculature is irregular, leaky, and unevenly distributed, producing regions of hypoxia and low blood flow. This perfusion heterogeneity limits radionuclide delivery and produces spatially variable radiopharmaceutical uptake independent of tumour viability [61]. Reduced perfusion has been demonstrated to diminish the uptake of multiple PET tracers including ^18^F-FDG, ^18^F-FMISO, and ^68^Ga-PSMA-targeted ligands, showing that reduced perfusion correlates with low tracer accumulation in hypoxic tumour areas. Similarly, in prostate and lung cancer imaging, ^68^Ga-PSMA-11 and ^18^F-FDG uptake have been shown to correlate with regional blood flow, underscoring the dependence of tracer delivery on vascular supply [62,63,64,65]. From a quantitative perspective, several PET–perfusion studies indicate that relatively small reductions in blood flow can already translate into substantial relative reductions in tracer accumulation, highlighting that microenvironment-driven variability in perfusion and fibrosis can exert effect sizes large enough to confound standard uptake-based response measurements. Defining microenvironmental “cut-offs”—for example, perfusion or fibrosis levels beyond which uptake no longer scales linearly with viable tumour burden—will be important to refine both imaging readouts and dosimetric models in future radiotheranostic trials.

Another consideration in RPT development is isotope selection for TME targets, this requires matching radionuclide decay properties to TME challenges, beyond target expression and uptake [66,67,68]. High-LET α-emitters (range ~50–100 µm; dense ionization) perform best when directly bound to tumour cells, delivering a high local dose while bypassing O_2_-dependent DNA repair. Preclinical studies show α-emitters trigger some bystander and immunogenic effects [66]. However, TME targeting using α-emitters may fail to deliver radiation effectively due to distance from tumour cells—though bystander effects can help compensate. Their short range minimizes off-target toxicity when lesion specificity holds, but poor penetration remains the key bottleneck, especially in fibrotic, poorly vascularised regions [66]. In contrast, β-emitters (range 1–10 mm; lower LET) use crossfire to cover uneven uptake in larger, well-perfused tumours with dense targets, though they raise bone marrow toxicity risks tied more to RPT distribution than emission type [66]. Microdosimetry models confirm TME factors like flow variation, high pressure, and ECM stiffness alter activity spread inside tumours: α-emitters perform better in low-flow zones whilst β-emitters perform better in bigger accessible volumes [68]. Bulk RNA-sequencing studies in preclinical brain tumours reveal α-emitters cause greater cell death versus β-emitters, upregulating pro-inflammatory pathways, cytokine expression, and immune activation, and they also provide greater anti-tumour immunity and synergy potential [69]. The development of TME-based guidelines incorporating clear criteria for isotope selection and dosimetry and trials grouped by microenvironment would be vital to ensure that the most appropriate strategies are being utilized. By theranostically addressing these delivery limitations and considerations directly within the TME, radiotracers provide a mechanistic path to improving treatment efficacy in tumours that are otherwise resistant to systemically delivered therapies. This opportunity to utilize the TME as a target and develop strategies to reduce its impact as a barrier through tracer development is one of the main reasons why theranostics is expected to grow in relevance in the coming decade.

### 3.2. Off-Target Uptake and Background Signal

Even when adequate tumour delivery is achieved, non-specific uptake and background signals in normal tissues remain major limitations, complicated further by the opposing pharmacokinetic requirements of diagnostic and therapeutic radiopharmaceuticals. Diagnostic radiopharmaceuticals require rapid tumour uptake, fast clearance from non-target tissues, and minimal retention to maximize the signal-to-noise ratio and lesion detectability [70]. In this setting, any off-target binding or slow washout reduces quantitative accuracy and image contrast. Conversely, RPT requires longer retention to deliver cytotoxic radiation doses, allowing a certain increase in background exposure to excretory organs (liver/kidneys) and an elevated hematological toxicity risk to ensure adequate radiation doses are received in the tumour [71,72]. Thus, implementing patient-specific voxel-level BED calculations becomes essential to balancing therapeutic radiation delivery with the protection of critical organs, ultimately defining the clinical success of RPT [73]. This divergence in required properties can make it challenging to develop theranostic agent pairs as often the target dictates the pharmacokinetic properties.

Specifically, targeting of stromal markers introduces unique challenges regarding off-target binding or background signals due to their frequent expression in normal tissues undergoing repair or inflammation, raising concerns about on-target, off-tumour toxicity. This is particularly critical for RPT, where absorbed radiation doses to healthy stromal-rich organs (e.g., lungs, liver, or wound-healing sites) could limit the safe administered activities and overall therapeutic index. Additionally, careful consideration of the exact stromal populations targeted by RPT is needed, given that some stromal subtypes play vital roles in tumour suppression whilst other pro-tumour CAFs have been shown to protect the tumour from immune attack and therapeutic damage [30]. This highlights the complexity of targeting stromal elements, where disrupting protective subtypes risks impairing anti-tumour immunity, while sparing pro-tumour populations may undermine RPT efficacy, underscoring the need for further understanding of stromal heterogeneity.

Similar challenges apply when targeting immune cells—ubiquitous marker expression in healthy tissues risks on-target off-tumour toxicity, radiation exposure to immune-rich organs (e.g., bone marrow, spleen) limits administered activities through myelosuppression, and precise subset selection is required given the contradictory roles of cytotoxic versus immunosuppressive populations (e.g., Tregs, MDSCs). Extensive dosimetry remains essential to truly understand the impact, as with the stroma [74]. However, immune targeting in RPT presents unique considerations, including prolonged lymphodepletion with reduced infection-fighting white blood cells, and systemic immune anti-tumour responses dependent on antigen release and type I interferon signalling and the need to combine with checkpoint inhibitors to counteract compensatory immunosuppression [75]. Flow cytometric-derived understanding of peripheral immune subsets is therefore essential to mitigate the risk of cytokine release syndrome following regulatory T cell ablation.

To minimize off-target uptake, several strategies have been explored. One such approach is the use of the increased permeability of tumour-associated neo-vasculature, which is referred to as passive targeting. This strategy allows radiopharmaceuticals or nanocarriers to penetrate cancerous regions and accumulate selectively in tumours with compromised drainage and high vascular permeability [76]. Another approach taking advantage of the features of the TME is using a stimulus-responsive release system. This method allows the regulated release of the active compound in response to tumour-specific conditions like an acidic environment (pH), hyperthermia (temperature) or higher levels of reactive oxygen species (redox-sensitive) [77]. Using the TME for delivery and targeting of radiopharmaceuticals is enhancing imaging precision as well as therapeutic efficacy by minimizing side effects through limiting off-target toxicity [77,78,79]. Improving normal-tissue sparing is essential for scalability, and advances in these strategies will be one of the key factors of why next-generation radiotheranostics can be implemented in clinical oncology.

### 3.3. Pharmacokinetics and Clearance

Radiopharmaceuticals follow the fundamental pharmacokinetic principles of administration, distribution, metabolism, and excretion (ADME), but in TME radiotheranostics, these processes are strongly altered by the stromal architecture, vascular dysfunction and inflammatory composition. Systemic distribution depends primarily on plasma protein binding, tissue permeability, and target affinity [77], but the TME is further restricted by abnormal perfusion, elevated interstitial fluid pressure, and ECM density.

Following systemic distribution, target accumulation occurs via specific molecular interactions within the TME, whether directed toward cancer cells or stromal components, while rapid plasma clearance enhances tumour-to-background contrast within the radionuclide’s physical half-life [77,80]. For oncologic imaging, renal clearance with urinary excretion is typically preferred, as hepatobiliary routes and slow intestinal transit can produce high abdominal background activity and hinder lesion detection.

Balancing these pharmacokinetic properties is essential for TME-oriented clinical intent. Diagnostic imaging benefits from fast tracer washout to reduce stromal and vascular background, whereas therapeutic radiopharmaceuticals require prolonged retention within tumour and stromal compartments to deliver effective cytotoxic doses [79,81]. Small molecules and peptides clear rapidly, primarily via glomerular filtration, which lowers background [79]. In contrast, antibodies and large protein constructs exhibit slow extravasation and extended circulation times, improving access to stromal targets but increasing whole-body radiation exposure [81]. To overcome these limitations, diverse chemical strategies (amino-acid substitutions, peptide cyclization, dimerization, PEGylation, albumin-binding motifs) have been implemented to improve serum stability and optimize biological half-life. In highly fibrotic or hypoxic TMEs, however, excessively prolonged circulation may impair imaging contrast and misalign the biological kinetics with the radionuclide’s physical decay constant. Short-lived isotopes suit fast-clearing ligands, whereas long-lived isotopes require slower biological turnover [79,81].

Internalization and intracellular trafficking further influence retention, particularly for ligands targeting stromal or immune components. Although receptor-mediated endocytosis enhances tumour uptake, radioactive catabolites may redistribute systemically [79]. Emerging AI-based modelling approaches are being explored to model biodistribution and TME heterogeneity; we hypothesize that these strategies will enable patient-specific optimization of tracer dosing and timing, strengthening the link between molecular imaging and precision radiotheranostics [77].

### 3.4. Radiation Dose and Toxicity Considerations

Radiotheranostic efficacy must be balanced against normal-tissue protection, as even well-targeted agents often accumulate in the kidneys, liver, salivary glands, and bone marrow, limiting administered activity [72,81,82]. This challenge is heightened when targeting TME processes that are not tumour-exclusive, such as fibrosis, since stromal markers are expressed in normal reparative tissues too. In peptide receptor RPT, for example, renal tubular reabsorption of radiolabelled peptides necessitates nephroprotective amino-acid infusions and individualized dosimetry [82], while hepatobiliary retention and salivary gland uptake similarly drive toxicity for lipophilic or PSMA-targeted agents [71,72]. Chemical strategies like modulating charge or lipophilicity using cleavable linkers, PEGylation, sugar conjugation, or reversible albumin binders, seek to reduce non-specific uptake, and emerging concepts such as pre-targeting or protease-cleavable masking aim to activate the radiopharmaceutical only within the tumour [71,72].

As toxicity is driven by radiation dose to normal tissues, patient-specific dosimetry is central to treatment planning. Radiopharmaceuticals are administered by activity (MBq) and evaluated by absorbed dose (Gy), integrating biodistribution, anatomy, and radionuclide properties [73]. Standard practice uses a tracer dose with serial imaging to derive time–activity curves and organ- or voxel-level dose estimates [83]. Yet, absorbed dose alone does not predict response. TME features including perfusion, hypoxia, fibrosis, ECM density, and DNA repair capacity, strongly modulate radiosensitivity [84]. Incorporating these variables into dosimetry is essential to estimate the biologically effective dose (BED) [85,86]. Hong et al. demonstrated this through spatial transcriptomics-integrated dosimetry modelling in prostate cancer, simulating ^177^Lu- and ^225^Ac-labelled PSMA, FAP, and GRPR ligands at voxel resolution [87]. Their findings—that identical macroscopic doses yielded divergent biological effects due to TME heterogeneity—highlight why current average-dose metrics often fail, underscoring the urgent need for more advanced, spatially resolved dosimetry studies to guide patient-specific RPT dose optimization [87]. Radiotheranostics balance efficacy against normal-tissue toxicity (kidneys, liver, glands, marrow), limited by TME overlap and requiring patient-specific dosimetry incorporating perfusion/hypoxia—key for regulations setting safe activity limits per IAEA/EANM standards [88,89,90].

## 4. Radiotheranostics in the Context of the TME

This section examines how radiotheranostics can address TME barriers through targeted agents (Table 1). The FAP and PD-L1 radioligands represent the most clinically advanced TME targets, offering therapeutic indices approaching PSMA/SSTR2 benchmarks, while collagen probes (CBP8, fluoroproline, collagelin) enable precise fibrosis phenotyping with therapeutic potential. The following subsections detail these leading candidates and their TME-specific applications.

### 4.1. Radiopharmaceuticals as Modulators of the TME

Recent advances in ^177^Lu-labelled radiopharmaceuticals not only demonstrate direct cytotoxic effects on tumour cells in neuroendocrine tumours (NETs) and metastatic castration-resistant prostate cancer (mCRPC) but also reveal vital important interactions—both directly through the TME (see Table 1). These effects occur both directly through TME targeting and indirectly via immune modulation and impacts on stromal components. RPTs can achieve therapeutic effects on the TME by targeting expressing tumour cells and the surrounding ECM and immune cells, both directly and indirectly [105,106]. In this section we primarily discuss ^177^Lu-DOTATATE and ^177^Lu-PSMA-617 due to their demonstrated clinical efficacy and well-characterized therapeutic mechanisms in NETs and mCRPC, respectively. Yet, despite growing interest in TME-targeted radiotheranostics, the dynamics of the TME before and after RPT remain comparatively underexplored, particularly when contrasted with the extensive literature on the TME in external beam radiotherapy.

An example of an RPT that both influences and is influenced by the TME is ^177^Lu-DOTATATE, which has significantly improved progression-free survival (PFS) in NET patients [107,108]. In gastroenteropancreatic-NET patients, the immune composition of the TME—specifically the low baseline densities of Tregs and CD8^+^TIM-3^+^ cells—was associated with improved therapeutic efficacy of ^177^Lu-DOTATATE [109]. Furthermore, studies in mouse models of NETS found ^177^Lu-DOTATATE treatment induced an anti-tumour immune response [110]. The study identified that ^177^Lu-DOTATATE treatment led to increased CD86+ expression (a proxy of anti-tumour CD8+ cells) suggesting that ^177^Lu-DOTATATE treatment leads to T cell activation [110]. Additionally, they also identified that death receptor FasL-expressing natural killer cells increasingly infiltrate the tumour leading to increased cell death [110]. This suggests that although the immunological consequences of RPT remain poorly understood, there appears to be great potential to modulate the TME and drive anti-tumour immunity even without direct immune targeting.

In prostate cancer, the most notable clinical advances are PSMA RPTs [111]. The VISION trial [112] demonstrated that compared against the typical standard of care, ^177^Lu-PSMA-617 significantly improved overall survival and imaging-based PFS in men with mCRPC [113]. Like ^177^Lu-DOTATATE, ^177^Lu-PSMA-617 therapy outcomes are affected by the TME; transcriptomic profiling of the TME in mCRPC patients treated with ^177^Lu-PSMA-617 revealed prognostic markers [114]. These include a favourable M1/M0 macrophage ratio, which was associated with improved overall survival across cohorts and additionally they found that PD-L2 expression predicts worse outcomes in treatment-naïve biopsies [114]. However, it shows protective effects post-treatment initiation. These findings suggest potential interaction with immune-checkpoint inhibition [114]. Additionally, hypoxia has been suggested to downregulate PSMA expression by suppressing VEGF-mediated angiogenesis; consequently, hypoxia-PET tracers such as ^18^F-FAZA and ^64^Cu-ATSM have been integrated into patient selection and therapeutic planning [115,116]. While promising, these findings illustrate the dynamic interplay wherein TME factors like hypoxia downregulate PSMA expression, while PSMA RPT itself upregulates PSMA via DNA damage response and reshapes immune dynamics—directly influencing patient outcomes and requiring prospective validation to optimize personalized therapy.

To date, checkpoint inhibitor therapy has shown efficacy only in biomarker-defined subsets (e.g., tumours with mismatch repair deficiencies or high tumour mutational burdens) [117], in which the microenvironmental and genomic context determines therapeutic responsiveness. However, recent evidence suggests that combining ^177^Lu-RPT (targeting PSMA) with immunotherapy achieves better efficacy than monotherapy by potentially converting immunologically “cold” tumours with poor T cell infiltration into “hot”, T cell-infiltrated phenotypes [118]. Moreover, PD-L2 expression has been shown to be prognostic in prostate cancer patients receiving PSMA-targeted therapy, emphasizing the role of immune escape mechanisms in determining RPT efficacy [114,119]. Consequently, combination approaches—such as pairing alpha- or beta-emitting RPTs with immune-checkpoint blockades or DNA damage response inhibitors—are now being actively explored to further improve patient outcomes [120].

### 4.2. Targeting TME for Theranostics

**Figure 2:**
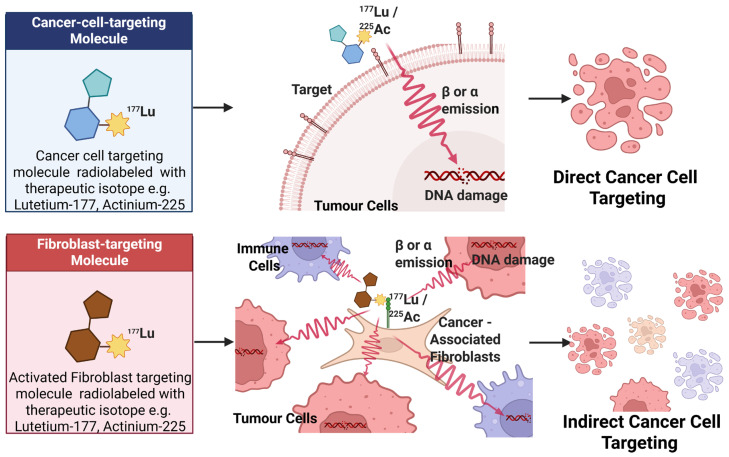
Conceptual strategies for ^177^Lu- and ^225^Ac-RPT: direct targeting of cancer cells versus indirect targeting of activated CAFs in stroma. Created in BioRender. Pandya, K. (2026) https://BioRender.com/6vp8z66, accessed on 22 February 2026.

Most clinically established RPTs currently target tumour-specific membrane receptors that internalize radiolabelled ligands, such as PSMA and somatostatin receptor 2 [121,122]. In the context of RPT, TME-directed strategies primarily target stromal fibroblasts, ECM components, or immune cells. However, their therapeutic efficiency may be lower than that of tumour cell-directed approaches, as the radiation dose is largely determined by the spatial proximity of the target to the malignant cells (Figure 2). Despite these challenges, several TME-associated targets have shown considerable promise for both diagnostic imaging and therapeutic applications [123].

Among stromal targets, FAP exemplifies both the potential and the limitations of TME-directed RPT [124]. FAP is highly expressed in activated CAFs across a wide range of epithelial tumours, sarcomas and granulation tissues and is relatively low in healthy tissue [125,126]. This upregulation in tumours allows the FAP inhibitor (FAPI) tracers to yield high tumour-to-background contrast in imaging [127]. Although FAP expression is often restricted to stromal fibroblasts rather than directly to target tumour cells, crossfire radiation from the stromal compartment can still deliver irradiation to adjacent malignant cells. However, despite a great deal of research for both FAP imaging and therapy, the current clinical impact of FAPI-PET remains limited. Furthermore, FAP presents a challenging target in patients with multiple comorbidities, as ubiquitous stromal remodelling occurs in response to diverse pathologies (e.g., fibrosis, wound-healing). These circumstances increase the likelihood of on-target, off-lesion toxicity, further limiting the safe administered activities and therapeutic index. As described previously, this is not only the case for stromal markers but also immune targets, which can be present systemically in many comorbidities.

Similarly, integrins mainly play a role in binding and forming a physical connection of cells to the ECM [128]. The αvβ3 integrin expressed on the luminal surface of neo-vasculature has been tested as a molecular target for imaging and therapy, which was upregulated on activated endothelial cells during tumour proliferation and vascularisation [129,130]. Beyond αvβ3, other integrins such as αvβ6 also show promise in tumours characterized by strong stromal activation and epithelial–mesenchymal transition. The αvβ6 receptor represents a promising biomarker and therapeutic target in highly aggressive and fibrotic tumours such as PDAC due to its selective over-expression [131,132]. However, integrin targeting faces significant hurdles: low receptor density or heterogeneity in expression on the endothelium limits radioligand accumulation and crossfire efficacy.

Collagen targeting has emerged for imaging TME fibrosis and stromal remodelling. Excessive type I collagen deposition characterizes diseases such as idiopathic pulmonary fibrosis and pancreatic ductal adenocarcinoma (PDAC), where it acts as a physical barrier to drug penetration and immune-cell infiltration [133,134]. The ^68^Ga-CBP8 enables non-invasive PET quantification of tumour-associated fibrosis and monitors treatment-induced stromal changes in PDAC models [21]. However, therapeutic translation remains challenging: collagen’s structural role spans physiological repair and pathological fibrosis, but low turnover rates may limit RPT efficacy, and clinical dosimetry data for collagen-directed radionuclides are lacking.

Finally, PD-L1 immune-checkpoint-targeting ^89^Zr-atezolizumab represents a TME-driven RPT which disrupts immunosuppression while delivering radiation [35,42]. This tracer has demonstrated heterogeneous tumour uptake in NSCLC, bladder cancer, and TNBC, often outperforming IHC for predicting response [97]. High spleen and lymphatic signals reflect physiological expression on antigen-presenting cells [97]. However, significant limitations constrain clinical utility: ubiquitous baseline PD-L1 on immune cells risks myelosuppression and lymphodepletion; low cell-surface density limits radioligand accumulation and crossfire; intra-tumoural heterogeneity complicates dosimetry; and compensatory Treg/MDSC expansion may undermine durable efficacy. Therapeutic translation remains preclinical, necessitating combination checkpoint strategies to mitigate infection risks.

In summary, TME-directed RPTs broaden the therapeutic landscape beyond tumour cells but remain limited by heterogeneous expression, systemic background, and insufficient crossfire dose delivery. These factors constrain both efficacy and safety, particularly in fibrotic or immune-active tissues. However, to ensure that TME-targeted radiopharmaceuticals are theranostically valuable over the next decade, we will require refined dosimetry, combination strategies, and smarter vector design to harness the TME’s therapeutic potential without compromising selectivity.

## 5. Innovative Approaches to Develop TME-Responsive Theranostic Tools

**Figure 3:**
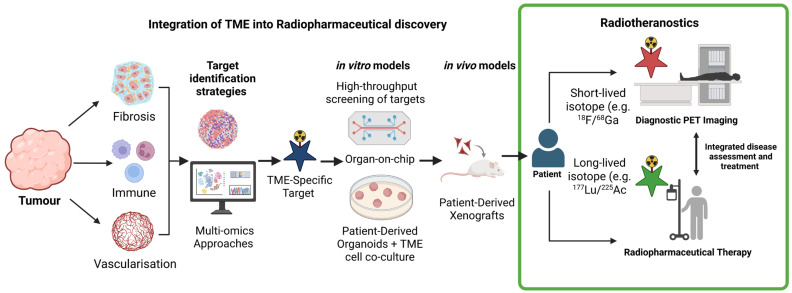
Workflow integrating TME biology, patient-derived organoids (PDOs), organ-on-chip systems, multi-omics analysis, and preclinical animal studies into radiotheranostic development to enable disease-specific assessment and individualized therapeutic strategies. Created in BioRender. Pandya, K. (2026) https://BioRender.com/8el98te, accessed on 22 February 2026.

The TME exerts a critical influence on tumour progression, treatment response, and resistance to therapy. In radiotheranostics, there is a growing need for experimental platforms that capture this complexity and enable the development of agents that are responsive to TME-specific features. This underscores a pivotal translational bottleneck: most pharmaceuticals and radiopharmaceuticals fail to progress from preclinical to clinical settings, primarily because standard models inadequately recapitulate the TME. This section outlines the emerging approaches, including spatial and single-cell omics, organ-on-chip systems, patient-derived organoids, and patient-derived xenografts, that can be integrated into a workflow for the rational design and preclinical evaluation of TME-informed theranostic strategies (Figure 3).

### 5.1. Spatial and Single-Cell Omics for Target Discovery of Radiotheranostics

Single-cell transcriptomics technologies—including single-cell RNA sequencing (scRNA-seq), spatial transcriptomics, and single-cell DNA sequencing (scDNA-seq)—enable high-resolution profiling of gene expression and metabolic states at the individual cell level [135]. By capturing tumour and microenvironmental heterogeneity, these approaches facilitate the identification of novel theranostic targets and allow for high-resolution prediction of post-treatment responses across tumour and TME cell types. Integrating such molecular data with mechanistic modelling further enhances our understanding of RPT dynamics. For example, studies have integrated spatial transcriptomics data from prostate cancer tissues with PBPK and convection reaction diffusion (CRD) modelling to simulate dosimetry and cell survival for ^177^Lu-PSMA and other RPTs labelled with either ^177^Lu or ^225^Ac [87]. This integration revealed distinct spatial dose–response patterns by mapping gene expression profiles, hypoxia signatures, and local cell-type composition, showing that FAP-targeted RPT was less effective in tumour-cell-dense regions, while ^225^Ac-labelled agents achieved higher efficacy in hypoxic or stromal-rich zones [87]. Such simulations are particularly valuable given that tumour target expression often correlates with differentiation status. In prostate cancer, for instance, some patients exhibit low PSMA expression and consequently poorer outcomes [136]. Moreover, PSMA expression may decline over the course of RPT, which potentially mimics a treatment response rather than loss of target availability [137].

Beyond assessing known targets, transcriptomic profiling also uncovers new theranostic opportunities. For example, spatial transcriptomics was used to identify and validate the cell-surface protein CLDN4 as a novel theranostic target based on its specific expression within the complex TME [138]. Transcriptomics approaches are therefore an ideal tool to accelerate RPT development. Similarly, Kraya et al. carried out a large-scale gene expression analysis identifying that FOLH1 (gene encoding PSMA) is upregulated in several non-CNS tumours with corresponding radiotracer uptake, supporting the expansion of PSMA-targeted imaging and therapy beyond prostate cancer [138,139].

Over the next decade, scRNA-seq, spatial transcriptomics, and scDNA-seq have the potential to advance theranostics across diverse diseases by characterizing tumour and TME heterogeneity to support target identification. Their integration with PBPK/CRD modelling could improve spatial dosimetry predictions and RPT response forecasting in varied clinical contexts.

### 5.2. Organ-on-Chip Systems

Organ-on-chip (OoC) systems are microfluidic platforms that culture cells in distinct 3D compartments to mimic tissue-level physiology. Using human-derived cells and tissues, they provide superior predictions of drug efficacy, toxicity, and pharmacokinetics compared to traditional 2D cultures. Moreover, OoC systems can replicate some parameters in TME, allowing precise control of parameters such as pH, oxygen distribution and hypoxia, ECM components and a universal medium for all co-cultured cells. Recent advances showed that microfluidic dynamics with integrated optical sensors are capable of continuously monitoring key physicochemical parameters such as pH and dissolved oxygen, thereby maintaining a precisely controlled microenvironment for long-term organoid or cell culture [140].

For RPT, OoC offers a useful tool to replicate stromal tumour interactions and study radiopharmaceutical transport, retention, and radiobiological effects (RBE) under physiologically relevant conditions [141,142]. Moreover, radiopharmaceutical OoC platforms facilitate studies of physiologically based pharmacokinetic (PBPK) modelling that simulate in vivo pharmacokinetics and dose distribution with high accuracy [141]. Furthermore, microfluidic radio assays have shown that cellular PBPK can be effectively modelled, supporting the application of on-chip PBPK modelling for quantifying RPT dose heterogeneity [143].

Over the next decade, OoC systems could significantly shape theranostics by offering human-relevant platforms to better predict radiopharmaceutical dosimetry, tumour–stromal dynamics, and patient responses in preclinical settings. This potential may accelerate target validation, lower RPT development risks through PBPK integration, and support more tailored treatment approaches via controlled microenvironment modelling.

### 5.3. Patient-Derived Organoids (PDOs)

PDOs represent the next step in model complexity and enable the testing and optimization of new radiotheranostic approaches. They can be directly established from patients’ tumour samples, making them patient-specific models for in vitro drug/radiopharmaceutical screening and enabling personalized cancer therapies [144,145]. Recent studies demonstrate that PDOs can retain key immunological features of the original tumour when generated using immune-preserving culture systems, enabling functional interactions with autologous TILs [146].

Tumour phenotypes and gene expression are highly heterogeneous both across patients and within individual tumours, impacting the efficacy of RPT for NETs and prostate cancer [147]. The recent establishment of GEP-NEN organoids that retain the genetic and phenotypic complexity of the original neuroendocrine carcinomas highlights the potential of PDOs to model tumour-intrinsic features and aspects of TME interactions relevant to RPT sensitivity and resistance [148]. As for mCRPC patients, the expression of PSMA and biological phenotypes are also distinct, which influence responses to ^177^Lu- and ^225^Ac- RPT [132]. In vitro studies have shown that 3D LNCaP tumour spheroids exhibit enhanced treatment responses compared with 2D monolayer cultures, likely due to differences in absorbed radiation dose and 3D cellular architecture [149].

Over the next decade, PDOs may enable precision theranostics by bridging patient tumours directly to preclinical high-throughput RPT testing, capturing individual heterogeneity that conventional models miss. This patient-centric approach could streamline target selection and refine dosing protocols, ultimately improving clinical translation for heterogeneous cancers.

### 5.4. Patient-Derived Xenografts (PDX)

Finally, in vivo preclinical models play a vital role in the assessment and translation of radiotheranostics agents. In this context, PDXs are essential for translational research in both imaging and therapeutic radiopharmaceutical development [150]. Like PDOs, PDX models are established directly from freshly resected patient tumour tissue but engrafted into immunocompromised mice [151]. This preserves human tumour components, making them widely used to evaluate the biodistribution and therapeutic efficacy of novel radiopharmaceuticals [152,153,154].

A major advantage of PDX models is the flexibility of implantation site. Tumour tissue can be implanted heterotopically, subcutaneously or orthotopically to capture PDX models to capture different aspects of the TME, including site-specific stromal interactions, vascularisation, and metastatic niches [155]. This makes PDXs a valuable tool for studying how the TME influences tumour growth, progression, and response to therapy.

However, despite these advantages, some limitations exist. While initially preserved, secreted factors and human immune components often diminish after 2–5 passages in immunodeficient hosts [150,156,157]. Critically, preclinical PDX models often fail to fully represent human disease complexity, particularly due to differences in TME composition, immune microenvironments, species-specific biology, and experimental design. For instance, many murine xenograft models are immunodeficient, lacking stromal and immune interactions critical for radiotracer uptake and biodistribution in humans [158]. Interspecies pharmacokinetic and dosimetry differences further complicate clinical translation.

Over the next decade, PDX models may refine theranostic translation by validating radiopharmaceutical efficacy across orthotopic sites and TME contexts that simpler models cannot replicate. Their ability to maintain patient-specific tumour architecture—despite passage-related limitations—could bridge critical gaps between preclinical data and personalized clinical dosing strategies.

Given that most theranostic agents are developed using preclinical methodologies with currently limited direct influence on clinical radiotheranostic agents, we believe improving these platforms to better incorporate TME complexity—through multiscale tools spanning single-cell transcriptomics, OoCs, PDOs, and PDXs—provides a framework linking molecular characterization with complex models. By addressing spatial heterogeneity, stromal interactions, and immune context, they may enhance target identification, refine dosimetry predictions, and strengthen predictive value in future development pipelines. Systematic TME integration offers the potential to generate more selective, biologically relevant theranostic agents tailored to individual tumour biology.

## 6. Future Directions in Microenvironment-Driven Radiotheranostics

As highlighted throughout this review, there is a growing understanding of the impact of the TME in radiotheranostics. It is seen not only as a barrier to effective radiopharmaceutical delivery but also as an opportunity to rethink and refine how radiotracers and therapeutics are developed. As described earlier, each component of the TME can influence radiotracer accuracy and therapeutic efficacy. Incorporating these microenvironmental factors into radioligand design represents the next logical step in advancing precision oncology. An example of this direction includes the development of ligands, delivery systems and combination therapies of radioligand and conventional drugs that respond to TME-specific features such as hypoxia, dense stroma, or altered metabolic conditions described previously.

An emerging field that could help shape TME-orientated theranostics is the integration of dosimetry with patient-specific transcriptomic and imaging data, which might offer a pathway to truly individualized RPT. As demonstrated through spatial transcriptomics, RPT responses may differ across tumour regions despite similar absorbed doses, driven by local microenvironmental factors such as hypoxia, stromal density, and immune niches [87]. Image feature extraction has been used for several clinical questions like the prediction of response in prostate cancer patients receiving PSMA-targeted therapy [159]. Potentially, image features and dual-tracer approaches can further enable quantification of fibrosis, immune infiltration, and receptor heterogeneity. An early example is the multi-tracer approach of imaging glucose metabolism and PSMA expression by PET, which could identify prognostic biomarkers in the context of PSMA therapy, though the clinical relevance of this dual PET phenotyping is not clear [160,161]. Meanwhile, studies such as the spatial-transcriptomics-guided development of tight-junction PET tracers in pancreatic cancer illustrate how molecular maps can directly inform radioligand design [138]. This framework may set the foundation for dynamic, microenvironment-aware theranostic algorithms that integrate TME features directly into therapeutic decision-making.

It is also plausible that future approaches could combine RPT with treatments that modify the TME, improving penetration into areas that are otherwise difficult to reach. Combining radiotheranostics with agents that remodel the microenvironment such as matrix-targeting therapies can be utilized in tandem with RPT. An example of this is PEGylated recombinant human hyaluronidase (PEGPH20), which degrades hyaluronan to improve tumour penetration and has been incorporated into nanomedicine strategies to enhance drug delivery and efficacy and has already been used in combination with chemotherapies, providing an opportunity for treatments like RPT [162]. Similarly, vascular-normalizing strategies, or drugs that alter immune-cell composition, may increase the accessibility of radiopharmaceuticals and sensitize resistant tumour niches. A recent example is ^177^Lu-DOTA-ATPS, an anti-ATP synthase monoclonal antibody labelled with ^177^Lu, which targets tumour-associated endothelial cells [163]. In a gastric cancer model, it produced both anti-angiogenic activity and targeted radiotherapeutic effects, illustrating how microenvironment-directed mechanisms can strengthen the impact of RPT [163]. However, disrupting processes such as ECM crosslinking or angiogenesis can also affect healthy tissues, and clinical studies have reported adverse effects including thromboembolism and muscle spasms [164,165]. Despite these challenges, careful evaluation of TME-modulating strategies may provide important opportunities to enhance the effectiveness of future RPT combinations.

Collectively, these advances point toward a future in which theranostics are not only target-directed but also microenvironment-aware, designed with explicit consideration of stromal biology, receptor heterogeneity, and tumour architecture. This shift has the potential to improve both tumour uptake and therapeutic durability across diverse solid malignancies.

## 7. Conclusions

TME features such as fibrosis, abnormal vasculature, heterogeneous stromal activation, and immune exclusion can hinder the delivery of radiopharmaceuticals, yet they also present potential targets. The available preclinical methods facilitate RPT development and could enable an increased speed of development of new theranostic approaches. Therefore, in the next decade, radiotheranostics offers a unique advantage to advance precision oncology: the very molecular and structural characteristics that restrict drug penetration can be visualized, quantified, and potentially leveraged to guide and enhance treatment.

## Figures and Tables

**Table 1:** TME-targeted radiopharmaceuticals.

Target	Example Radiopharmaceuticals	Precursor Format	Application	Refs.
Fibrosis- and ECM-related targets				
FAP	^68^Ga-FAPI-04; ^177^Lu-FAP-2286	Inhibitor	Theranostics	[24]
Collagen	^68^Ga-CBP8	Peptide	Imaging	[21]
Tenascin-C	^18^F/^64^Cu-FB-tenascin-C aptamer; ^131^I-Tenarad	Aptamer, antibody	Theranostics	[25,26]
Fibronectin	^64^Cu-NJB2; ^131^I-L19SIP	Antibody	Theranostics	[27]
Angiogenesis-related targets				
Integrin (e.g., αvβ3, αvβ6, αvβ8, α5β1)	^68^Ga-NODAGA-RGD; ^177^Lu-AB-3PRGD_2_	Peptide	Theranostics	[55,91,92]
VEGF	^89^Zr-Bevacizumab; ^177^Lu-DOTA-VG76e	Antibody	Theranostics	[56]
PSMA	^68^Ga-PSMA-11; ^177^Lu-PSMA-617	Inhibitor	Theranostics	[93,94]
Immune-related targets				
PD-1	^89^Zr-nivolumab; ^177^Lu-αPD-1	Antibody	Theranostics	[95,96]
PD-L1	^89^Zr-atezolizumab; ^177^Lu-DOTA-Y003	Antibody	Theranostics	[35,42,97]
CTLA-4	^89^Zr-ipilimumab	Antibody	Imaging	[98]
CD8	^89^Zr-Df-IAB22M2C	Antibody	Imaging	[37]
Granzyme B	^68^Ga-NOTA-GZP; ^68^Ga-grazytracer	Peptide	Imaging	[99,100]
IFN-γ	^89^Zr-DFO-anti-IFNγ	Antibody	Imaging	[40]
IL-2R	^68^Ga-interleukin-2	Protein	Imaging	[39]
TAMs (e.g., CD206, TSPO)	^68^Ga-Anti-CD206-sdAb	Antibody	Imaging	[41,101]
B7-H3	^89^Zr-DS-5573a; ^124^I/^131^I-8H9	Antibody	Theranostics	[102,103]
Hypoxia-related targets				
/	^18^F-FMISO	Nitroimidazole derivative	Imaging	[104]
CAIX	^89^Zr/^177^Lu-girentuximab	Antibody	Theranostics	[58,59]

## Data Availability

No new data were created or analyzed in this study. Data sharing is not applicable to this article.

## Abbreviations

The following abbreviations are used in this manuscript:

CAF	Cancer-associated fibroblast
DNA	Deoxyribonucleic acid
ECM	Extracellular matrix
FGF	Fibroblast growth factor
LOX	Lysyl oxidase
LOXLs	Lysyl oxidase-like proteins
mCRPC	Metastatic castration-resistant prostate cancer
MMP	Matrix metalloproteinase
NET	Neuroendocrine tumour
OoC	Organ-on-chip
PD-1	Programmed cell death protein-1
PD-L1	Programmed death-ligand 1
PDGF	Platelet-derived growth factor
PDO	Patient-derived organoid
PDX	Patient-derived xenograft
PET	Positron emission tomography
PSMA	Prostate-specific membrane antigen
RPT	Radiopharmaceutical therapy
SPECT	Single-photon emission computed tomography
TAM	Tumour-associated macrophage
TME	Tumour microenvironment
Treg	Regulatory T cell
VEGF	Vascular endothelial growth factor
